# Quality of life assessment in vitiligo patients: a cross-sectional study^☆^

**DOI:** 10.1016/j.abd.2026.501305

**Published:** 2026-03-27

**Authors:** Natália Maria Neves Simões, Gabrielle Cassulo Franciscatti, Luis Alberto Ribeiro Froes Junior, Maria Victória Quaresma

**Affiliations:** a Department of Dermatology, Hospital das Clínicas, Faculty of Medicine, Universidade de São Paulo, São Paulo, Brazil; b Faculty of Medicine, Universidade de São Paulo, São Paulo, SP, Brazil; c Department of Pathology, Faculty of Medicine, Universidade de São Paulo, São Paulo, SP, Brazil

Dear Editor,

Vitiligo, a chronic acquired depigmenting disorder, affects 0.5%–2% of the global population and, although typically asymptomatic, often leads to substantial psychosocial consequences, including stigmatization, diminished self-esteem, and social withdrawal.^1^, ^2^, ^3^ Multiple studies documented a higher prevalence of psychiatric comorbidities, particularly anxiety and depression, among vitiligo patients versus the general population.^3^, ^4^

While general dermatology instruments like the Dermatology Life Quality Index (DLQI) are widely used to assess disease burden, they may systematically underestimate vitiligo's specific psychosocial impact.^5^ These generic tools often emphasize physical symptoms such as pain or pruritus, largely irrelevant to vitiligo patients. To address these limitations, the Vitiligo-Specific Quality of Life instrument (VitiQoL)^6^ was developed as the first disease-specific questionnaire, offering targeted evaluation of emotional and social impairment unique to this condition. It was subsequently translated and validated in Brazilian Portuguese, ensuring cultural and linguistic appropriateness for the local population.^7^

Despite extensive international research on vitiligo's psychosocial impact, data from Brazilian referral centers remain remarkably scarce. This study aimed to comprehensively assess vitiligo patients' Quality of Life (QoL) using the VitiQoL instrument and systematically identify clinical and demographic variables associated with QoL impairment, emphasizing disease activity and progression ‒ dynamic factors often inadequately captured in cross-sectional assessments.

This cross-sectional study included 100 consecutive adult patients with clinically confirmed vitiligo, followed at the Dermatology Outpatient Clinic of Hospital das Clínicas, Faculdade de Medicina da Universidade de São Paulo. Inclusion criteria were age ≥ 18-years, functional literacy in Brazilian Portuguese, adequate cognitive ability to complete questionnaires, and written informed consent. Patients with concurrent pigmentary disorders or other disfiguring dermatological conditions were systematically excluded to avoid potential confounding effects on body image perception and quality of life assessment.

All participants completed demographic questionnaires capturing age, sex, marital status, and educational attainment, and underwent a comprehensive clinical evaluation by dermatologists. Vitiligo subtype was meticulously classified according to Vitiligo Global Issues Consensus Conference criteria.^8^ Fitzpatrick skin phototype^9^ was systematically recorded, and disease extent was measured using the validated Vitiligo Extent Score (VES)^10^ via online visual assessment tools. Disease progression was rigorously categorized into five groups: “improving”, “stable for less than two years”, “stable for more than two years”, “worsening slowly”, and “worsening rapidly”, based on a comprehensive evaluation of lesion dynamics and clinical activity markers, including Köbner phenomenon, trichrome patterns, and confetti macules.^8^, ^11^

QoL was assessed using the validated Brazilian Portuguese VitiQoL, a psychometrically robust 16-item instrument specifically designed for vitiligo patients.^7^ The VitiQoL ranges from 0 to 96, with higher scores indicating worse QoL, similar to the DLQI, whereas in other QoL questionnaires, such as the SF-36, AcneQoL, and WHOQOL-BREF, higher scores reflect better QoL. To minimize potential response bias and ensure participant privacy when reporting sensitive psychosocial aspects, all instruments were strictly self-administered. Clinical evaluation and VitiQoL application were performed on the same day to ensure data consistency.

Statistical analyses were conducted using STATA 11.0. Descriptive statistics were reported as means, medians, and 95% confidence intervals. Associations between VitiQoL scores and categorical variables were evaluated using the Kruskal-Wallis test, while Spearman's correlation coefficient was employed for continuous variables. A significance level of p < 0.05 was adopted for all statistical comparisons.

Study participants had a mean age of 50.9-years (SD = 16.0) and were predominantly female (71%) (Table 1). Generalized vitiligo represented the most common clinical form (76%), followed by acrofacial (11%), focal (7%), segmental (3%), indeterminate (2%), and mixed variants (1%). Most patients exhibited Fitzpatrick phototype IV (44%), consistent with Brazilian demographics. Mean disease duration was 17.8-years, highlighting vitiligo's chronic nature and sustained psychosocial burden. Regarding disease progression, 26% of patients were classified as “worsening rapidly” and 27% as “improving”, ensuring representation across the disease spectrum.

**Table 1:** Sociodemographic and clinical characteristics of the 100 vitiligo patients included in the study.

Variable	Category	n (%) or Mean ± SD
Age (years)	–	50.9 ± 16.0
Sex	Female	71 (71.0%)
	Male	29 (29.0%)
Marital status	Married	45 (45.0%)
	Unmarried	55 (55.0%)
Disease duration (years)	–	17.8 ± 14.5
Total body surface affected (%)	–	9.8 ± 15.2
Vitiligo type	Generalized	76 (76.0%)
	Acrofacial	11 (11.0%)
	Focal	7 (7.0%)
	Segmental	3 (3.0%)
	Indeterminate	2 (2.0%)
	Mixed	1 (1.0%)
Disease progression	Improving	27 (27.0%)
	Stable > 2 years	13 (13.0%)
	Stable < 2 years	18 (18.0%)
	Worsening slowly	16 (16.0%)
	Worsening rapidly	26 (26.0%)
Fitzpatrick phototype	II–III	36 (36.0%)
	IV–VI	64 (64.0%)

Data are presented as absolute numbers and percentages (n [%]) or as mean ± Standard Deviation (SD).

Mean VitiQoL score was 41.6 (maximum possible: 96). Female patients reported significantly greater QoL impairment than males (median: 43 vs. 32; p = 0.018), consistent with previously documented^2^ gender disparities in psychosocial impact (Table 2). Notably, married individuals demonstrated significantly worse QoL scores than unmarried participants (median: 51 vs. 37; p = 0.040). Although counterintuitive, this may reflect vitiligo's impact on intimate relationships, body image within partnerships, or concerns about partner acceptance. Importantly, the VES assessment tool does not separately evaluate genital involvement, which may partially explain this association and represents a limitation of current disease measurement instruments.

**Table 2:** Impact of vitiligo on patients' quality of life according to sociodemographic and clinical factors, measured using the VitiQoL instrument.

Variable	Category	Median VitiQoL [95% CI]	p-value
Sex	Female	43.0 [36.0; 55.5]	0.018
	Male	32.0 [13.7; 43.2]	
Marital status	Married	51.0 [36.2; 59.2]	0.040
	Unmarried	37.0 [28.8; 41.3]	
Fitzpatrick phototype	II–III	41.0 [26.5; 51.8]	0.427
	IV–VI	39.0 [28.2; 48.1]	
Disease progression	Improving	24.0 [13.9; 35.1]	<0.001
	Stable > 2 years	39.0 [6.7; 60.0]	
	Stable < 2 years	47.0 [21.6; 67.2]	
	Worsening slowly	37.5 [31.5; 42.5]	
	Worsening rapidly	59.0 [48.3; 71.7]	
Vitiligo type	Generalized	42.0 [35.9; 51.0]	0.604
	Acrofacial	27.0 [11.0; 54.1]	
	Focal	40.0 [9.5; 45.4]	
	Segmental	41.0 [0.0; 62.0]	
	Indeterminate	31.0 [27.0; 35.0]	
	Mixed	22.0 [22.0; 22.0]	

Data are presented as median values with 95% Confidence Intervals (95% CI).

QoL outcomes did not differ significantly across educational levels, skin phototypes, or vitiligo subtypes, suggesting these factors have minimal influence on psychosocial burden. Similarly, disease duration showed no correlation with VitiQoL scores (rho = -0.13; p = 0.180), indicating that chronicity alone is not a primary determinant of emotional distress (Table 3). In stark contrast, disease activity demonstrated a profound association with QoL (p < 0.001). Patients classified as “worsening rapidly” exhibited the highest mean VitiQoL scores (59), while those categorized as “improving” had the lowest scores (24). Intermediate categories demonstrated a logical progressive pattern: “stable < 2-years” (47), “stable > 2-years” (39), and “worsening slowly” (37.5).

**Table 3:** Correlation between VitiQoL scores and clinical or demographic variables using Spearman’s rank correlation coefficient (rho).

Variable	Spearman’s rho	p-value	Clinical interpretation
Disease duration (years)	−0.13	0.180	No significant correlation
Total body surface area (%)	0.27	0.006	Weak positive correlation
Trunk (%)	0.26	0.008	Weak positive correlation
Extremities (%)	0.29	0.003	Weak positive correlation
Hands (%)	0.24	0.014	Weak positive correlation
Face (%)	0.18	0.066	No significant correlation
Feet (%)	0.18	0.068	No significant correlation
Age (years)	0.02	0.836	No significant correlation

Values close to 0 indicate no significant correlation; values between 0.20 and 0.39 indicate a weak positive correlation.

VES-derived total Body Surface Area (BSA) involvement demonstrated a weak but statistically significant correlation with VitiQoL scores (rho = 0.27; p < 0.05). Anatomical region-specific analyses revealed weak correlations for trunk (rho = 0.26), extremities (0.29), and hands (0.24), but surprisingly, no significant association with facial or pedal involvement. The absence of correlation between facial vitiligo and QoL impairment was particularly unexpected, given the face's visibility and social significance. This finding warrants further investigation and may reflect adaptation mechanisms, age-related acceptance, or cultural differences in facial appearance perception.

The highest individual VitiQoL item scores related to self-perceived disease severity and concerns about disease progression, emphasizing the emotional distress associated with unpredictability and perceived loss of control. This finding strongly supports targeting disease activity ‒ rather than merely extent ‒ as a primary therapeutic objective.

These results demonstrate that vitiligo's impact on quality of life is more profoundly influenced by subjective disease perception and dynamic progression patterns than by objective clinical parameters such as extent or chronicity. Treatment decisions should therefore systematically incorporate patient-reported outcomes, with explicit emphasis on halting disease progression as meaningful therapeutic goals. These findings provide compelling evidence for including patient-reported outcome measures in both clinical trials and routine dermatological practice. The widespread assumption that vitiligo is cosmetically benign and requires minimal intervention may contribute to systematic undertreatment and preventable psychological harm. Study limitations include a cross-sectional design precluding causal inference and single-center recruitment, potentially limiting generalizability.

In conclusion, this study provides novel data from a major Brazilian referral center, demonstrating vitiligo's significant impact on quality of life, particularly among women and patients experiencing disease progression. Surface area involvement alone proves insufficient for guiding evidence-based therapeutic decisions. Clinicians should consider not only vitiligo's visible manifestations but also its evolving clinical course and profound psychological consequences. Early recognition, empathetic care, and proactive treatment interventions may substantially improve patient well-being and long-term outcomes.

## ORCID ID

Natália Maria Neves Simões: 0009-0004-6639-4521

Gabrielle Cassulo Franciscatti: 0009-0007-7216-0045

Luis Alberto Ribeiro Froes Junior: 0000-0002-1140-3046

Maria Victória Quaresma: 0000-0003-2891-1650

## Research data availability

The entire dataset supporting the results of this study was published in this article.

## Financial support

None declared.

## Authors' contributions

Natália Maria Neves Simões: Study conception and planning; data collection, analysis and interpretation; statistical analysis; preparation and writing of the manuscript; intellectual participation in propaedeutic and/or therapeutic management of studied cases; critical literature review; approval of the final version of the manuscript.

Gabrielle Cassulo Franciscatti: Preparation and writing of the manuscript; manuscript critical review; critical literature review; approval of the final version of the manuscript.

Luis Alberto Ribeiro Froes Junior: Manuscript critical review; approval of the final version of the manuscript.

Maria Victória Quaresma: Study conception and planning; manuscript critical review; intellectual participation in propaedeutic and/or therapeutic management of studied cases; effective participation in research orientation; critical literature review; approval of the final version of the manuscript.

## Conflicts of interest

None declared.
